# Contribution of riboflavin supply pathways to *Vibrio cholerae* in different environments

**DOI:** 10.1186/s13099-017-0214-9

**Published:** 2017-11-14

**Authors:** Andrés Fuentes Flores, Ignacio Sepúlveda Cisternas, José Ignacio Vásquez Solis de Ovando, Alexia Torres, Víctor Antonio García-Angulo

**Affiliations:** 1 Programa de Microbiología y Micología, Instituto de Ciencias Biomédicas, Universidad de Chile, Santiago, Chile; 2 Escuela de Biotecnología, Universidad Mayor, Campus Huechuraba, Santiago, Chile; 3 Facultad de Medicina Norte, Pabellón L. Santiago de Chile, ZIP 8380453 Santiago, Chile

**Keywords:** Riboflavin transport, Biosynthesis, *Vibrio cholerae*, Vitamin B2, Environmental fitness

## Abstract

**Background:**

The waterborne diarrheagenic bacterium *Vibrio cholerae*, cause of the pandemic cholera disease, thrives in a variety of environments ranging from estuarine waters to the human intestinal tract. This species has two ways to obtain the essential micronutrient riboflavin, de novo biosynthesis and environmental uptake through the RibN importer. The way these functions interrelate to fulfill riboflavin needs in different conditions in this species is unknown.

**Results:**

This study analyzed the contributions of riboflavin biosynthesis and transport to the culturability of *Vibrio cholerae* in river and seawater in vitro and in the *Caenorhabditis elegans* nematode host model. Elimination of the *ribD* riboflavin biosynthetic gene renders the bacteria riboflavin-auxotrophic, while a *ribN* mutant strain has no growth defect in minimal media. When growing in river water, deletion of *ribD* causes an impairment in culturability. In this condition, the ∆*ribN* strain has a defect to compete against a wild type strain but outcompetes the ∆*ribD* strain. The latter effect is inverted by the addition of riboflavin to the water. In contrast, growth in seawater causes a loss in culturability independent of riboflavin biosynthesis or transport. In the *C. elegans* model, only the ∆*ribD* strain is attenuated.

**Conclusion:**

Results indicate that while riboflavin biosynthesis seems to outweigh riboflavin uptake, the latter may still provide a selective advantage to *V. cholerae* in some environments.

**Electronic supplementary material:**

The online version of this article (10.1186/s13099-017-0214-9) contains supplementary material, which is available to authorized users.

## Introduction

Riboflavin, also named vitamin B2, is an essential micronutrient. This vitamin serves as precursor for biological electron transfer cofactors, mainly flavin mononucleotide (FMN) and flavin adenine dinucleotide (FAD). These products are required for several redox enzymatic reactions. The myriad of cellular cues that require flavins include carbon metabolism, oxidative stress protection, photosensitization and metabolism of other vitamins [1, 2]. To accomplish its riboflavin demands, many bacteria may use the riboflavin biosynthetic pathway (RBP) comprised by proteins encoded by the *ribDEABH* genes, while others lack the RBP but instead encode riboflavin importer proteins [3–10]. Frequently riboflavin biosynthesis and uptake abilities coexist in bacteria, but the way these two biological functions coordinate to provide riboflavin has been scarcely studied [6, 11–13]. Thus, while either riboflavin biosynthesis or uptake can be used to support life in many bacterial species, their relative importance in the so called riboflavin-opportunistic species [14] has not been established. It is conceivable that riboflavin uptake may help saving metabolic energy in a riboflavin prototrophic species and also it may provide riboflavin for specific cellular functions [5, 6, 12, 14, 15].

*Vibrio cholerae* is a pandemic pathogen causing cholera, an acute diarrheal disease responsible for up to 120,000 deaths every year worldwide [16]. This bacterium survives in fresh water and marine environments where it may form biofilms in biotic and abiotic surfaces and also grow in planktonic form [17–19]. After human consumption, *V. cholerae* colonizes and replicates in the host intestine where it expresses virulence factors, most notorious the cholera toxin and the toxin corregulated pilus, responsible for diarrhea and bacterial initial intestinal adhesion [20]. Given the wide spectrum of conditions that faces during its life cycle, *V. cholerae* likely must deal with variable nutrient availability. This organism is a riboflavin-prototrophic bacterium encoding a full RBP which features two putative *ribB* and *ribA* genes. In addition, it is also able to internalize riboflavin using the RibN importer [7, 21, 22]. In this species, the complexity of the RBP and its co-occurence with the riboflavin uptake ability may be related to the intrinsic variations of riboflavin concentrations featured by the wide spectrum of environments inhabitable to the pathogen. Thus, *V. cholerae* comprises an interesting case of study for the overlap of riboflavin provision pathways in bacteria. Recently, we reported that the presence of extracellular riboflavin downregulates the monocistronic gene *ribB*, while having no effect on the expression of the RBP genes in the main riboflavin biosynthetic operon nor on *ribN* [22]. Nonetheless, no study has assessed the role of the riboflavin provision pathways of *V. cholerae* in different environments. In this report, we started defining the individual contributions of riboflavin biosynthesis and of the RibN riboflavin importer to *V. cholerae* by assessing the fitness of ∆*ribN* and ∆*ribD* strains in a set of different conditions, namely seawater and river water microcosms and the *Caenorhabditis elegans* host model.

## Methods

### Bacterial strains, plasmid construction and growth curves

This work used WT *V. cholerae* N16961 and its *∆ribN* and *∆ribN*::*kan* derivative strains [22]. *V. cholerae* ∆*ribD* was constructed by homologous recombination using the same protocol as that for *ribN* mutants [22, 23] and primers ribDH1P1 (5′- ATGCCTATGTTTACCTCTTTTGATCATCAAATGATGTCTCGCGCGGTGTAGGCTGGAGCTGCTTC-3′) and ribDH2P2 (5′-CTAATCTTTTGTTTTTGGGGTGGCGATGATCCGCAAATCTGCCCCCATATGAATATCCTCCTTAG-3′). The eliminantion of the *ribD* gene was corroborated by PCR using primers nrdR Fw and ribE Rv [22] flanking the deletion. To construct plasmid pB1ribD, *ribD* from *V. cholerae* N16961 was amplified by PCR from genomic DNA and primers nrdR Fw and ribE Rv. The resulting PCR fragment was cloned into pGEM T Easy (Promega) according to manufacturer´s instructions to generate pGribD. Next, the fragment containing *ribD* was excised from this plasmid by ApaI and SpeI digestion and subcloned into the same sites of pBBR1 MCS 1. This construction was corroborated by sequencing. For growth curves in T minimal media [24], the indicated bacterial strains were grown overnight on LB plates at 37 °C. Then, they were inoculated in 5 ml fresh LB broth and incubated at 37 °C at 160 rpm until they reached an optical density (O.D.) of ~ 0.7. Next, cultures were centrifuged at 12,000 rpm, supernatant discarded and pellet washed twice with T media and resuspended in fresh T media. 100 µl of these cultures were used to inoculate 50 ml of T media or T media with different riboflavin concentrations (0, 0.01, 0.1, or 2 µM) as indicated in 125 ml flasks, incubated at 37 °C on an orbital shaker at 160 rpm and O.D. determined at the indicated time points.

### Culturability on seawater and river water assays

The indicated strains were grown overnight in LB plates. Next, a colony was inoculated in 5 ml of LB broth and growth at 37 °C at 160 rpm until a D.O. ~ 0.7. These cultures were washed twice and resuspended in sterile water. 50 µl were used to inoculate 50 ml of filter-sterilized water from Mapocho river or Valparaiso bay, Chile, in Erlenmeyer flasks. Flasks were incubated at 25 °C with soft shaking (50 rpm). Initial inocula were determined by serial dilution platting on LB plates and incubation at 37 °C for 18 h and culturability of the strains was assessed in the same way at the indicated time points. Importantly, *V. cholerae* is able to enter viable but non-culturable (VBNC) state [25, 26]. In this state, the metabolic activity is reduced in order to survive harsh environments and bacteria do not grow in subsequent transfers to rich media [26]. Thus, we thoroughly use the term “culturability” in reference to UFC counts, as this term includes metabolically active bacteria and excludes both VBNC and dead cells. For competition experiments, 25 µl of each strain obtained as those for single inoculations were added to the same flask. In such experiments, the ∆*ribN*::*kan* strain was used [22]. For initial inocula and also for each time point, serial dilutions up to 10^−6^ were plated in order to obtain isolated colonies. For each determination, 200 isolated colonies were replicated in T medium to determine the fraction of each strain in the culture. Colonies of the ∆*ribN* and ∆*ribD* strains were identified by testing for kanamycin resistance and riboflavin auxotrophy, respectively.

### Real time PCR

*Vibrio cholerae* WT was grown in 50 ml of T media at 37 °C and 160 rpm until D.O. = 0.7 and on 3 beakers with 50 ml Mapocho river water each until day 3 as described previously. 1 ml of T and 150 ml of river water were centrifuged, washed twice, RNA extracted with the Thermo Scientific Genejet RNA purification kit and digested with Turbo DNA-free DNAase according to manufacturer´s instructions. Next, complementary DNA was synthesized using AffinityScript QPCR cDNA Synthesis kit from Agilent Technologies. RT-PCR was performed using the Brilliant II SYBR Green QPCR Master Mix kit in a One-Step Applen Biosystems (Life Technologies) thermocycler. A reaction without reverse transcriptase was included for each sample in each run as a control. Relative expression in the indicated conditions was determined using the ∆∆Ct method as developed before [27]. Measurements were normalized using the 16s rRNA gene. The sets of primers used for RT-PCR were ribN Fw/ribN Rv for *ribN*, ribD Fw/ribD Rv for *ribD* and 16s Fw/16s Rv for 16s [22].

### *C. elegans* survival assays

6 cm Petri dishes with NGM medium [28] containing 100 µl of overnight cultures of *E. coli* OP50 or the indicated *V. cholerae* strains were incubated for 18 h at room temperature. Next, 30–32 L4 larvae of *C. elegans* strain N2 were added to each plate and incubated at 25 °C. The viability of the worms was scored at the indicated times by response to touch. Animals were transferred to fresh plates every 72 h to avoid progeny interference.

## Results

**Fig. 1:**
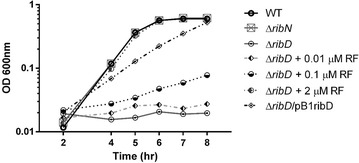
Growth curves of N16961 *V. cholerae* WT and its derivative strains in minimal T medium. The strains were inoculated in T media or T media plus riboflavin (RF) as indicated and incubated at 37 °C and O.D. measured hourly. The average of three independent experiments is shown

To start defining the individual contributions of riboflavin biosynthesis and of the RibN riboflavin importer to the fitness of *V. cholerae*, we evaluated the effect of *ribN* and *ribD* deletions in different conditions. First, the riboflavin requirements of these strains were evaluated on defined T minimal medium [24] (Fig. 1). Elimination of *ribN* produced no growth defects compared to the WT strain. However, disruption of riboflavin biosynthesis by the elimination of *ribD* prevented *V. cholerae* from growing in T medium. Addition of increasing amounts of riboflavin to the medium gradually restored the ability of the ∆*ribD* strain to grow. Addition of 0.01 or 0.1 µM riboflavin produced only slight increases in the growth capacity, while 2 µM riboflavin allowed the ∆*ribD* strain to grow to WT levels. This result reflex the ability of this strain to internalize riboflavin. Also, complementation of the ∆*ribD* strain with the multicopy plasmid pB1ribD encoding *ribD* from *V. cholerae* restored growth in the absence of exogenous riboflavin, although with a lower rate compared to the WT. The lack of full complementation could indicate that the genetic charge generated by the multicopy plasmid may be disadvantageous to the bacteria. Besides, the overproduction of highly reactive riboflavin biosynthesis intermediaries due to overexpression of RibD could affect growth. Riboflavin biosynthesis intermediaries may be toxic to the cell [29].

**Fig. 2:**
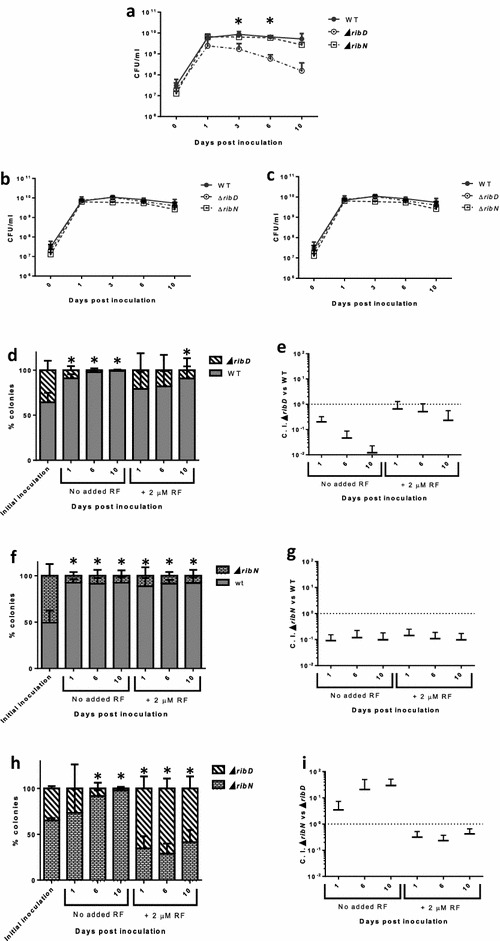
Contribution of *ribD* and *ribN* to *V. cholerae* growth in river water. Culturability of *V. cholerae* strains in filter-sterilized water from Mapocho river (**a**) and effect of 0.1 µM (**b**) or 2 µM (**c**) riboflavin (RF). Culturability of *V. cholerae* in experiments of co-inoculation of strains in water from the Mapocho river with or without 2 µM exogenous riboflavin and the same results expressed as competitive index (C.I.) for WT versus ∆*ribD* (**d**, **e**), WT versus ∆*ribN* (**f**, **g**) and ∆*ribD* versus ∆*ribN* (**h**, **i**). The average and standard deviation of three independent experiments is shown. *denotes statistically significant difference (P < 0.05) between WT and ∆*ribD* (**a**) or between the indicated bar and the initial inoculum (**d**, **f**, **h**) determined by t tests analysis

Next, we evaluated the fitness of these strains inoculated in river water. The WT strain showed an initial increase in colony forming units (CFU) numbers of approximately 2.5 log after the first day of incubation (Fig. 2a). This culturability level was thereafter maintained around the same until day 10. The ∆*ribN* strain showed a similar pattern of culturability. However, the ∆*ribD* displayed a gradual decrease in CFU numbers after 1 day of incubation. At day 10, CFU numbers were around 100-fold reduced for the ∆*ribD* compared to WT. On one hand, addition of riboflavin to water did not increase culturability of the WT and ∆*ribN* strains (Fig. 2b, c). On the other hand, the culturability defect displayed by the ∆*ribD* was abrogated by addition of 2 µM (Fig. 2c) or even 0.1 µM riboflavin to the water (Fig. 2b). Following, we performed competition experiments where two strains were co-inoculated. Competition assays between a mutant strain and its WT parental are usually performed in order to identify defects to colonize a niche. Competition assays are more sensitive than single strain assays as direct competition against a fully capable strain exacerbates colonization impairments. Also, testing two strains within the same experimental unit usually decreases variation among time points and replicates [30]. In these co-culture experiments, the percentage of each strain remaining as culturable colonies at different time points was determined. In line with the result showing a defect to grow in plain river water, the ∆*ribD* strain was gradually outcompeted by the WT strain from day 1 to day 10 (Fig. 2d, e). This outcome was delayed but not avoided by the addition of riboflavin to water. Meanwhile, the ∆*ribN* showed a defect to compete against the WT strain manifested from day 1 and maintained the same until day 10 (Fig. 2f, g). This effect was independent of added riboflavin. When the two mutants were co-inoculated (Fig. 2h, i), the ∆*ribD* was progressively outcompeted by the ∆*ribN*. Nonetheless, addition of riboflavin overturned this pattern, with the ∆*ribD* mantaining a small advantage to compete against the ∆*ribN* strain. Yet, this leverage was lesser than the advantage displayed by the ∆*ribN* over the ∆*ribD* strain without exogenous riboflavin. We previously reported that extracellular riboflavin does not changes the expression of *ribD* nor of *ribN* [22]. In order to evaluate if river water induces changes in the expression of these riboflavin supply pathways, we determined the relative expression of both genes in T medium versus river water. Results showed a reduction in expression in river water. This reduction was similar for *ribD* and *ribN*, with the expression in river water reduced to roughly one tenth of that in T medium (Additional file 1). To assess if growth in river water boosts the expression of any riboflavin provision pathway over the other, we also determined the *ribD*/*ribN* expression ratio in T medium and in river water. Results show that the ratio of *ribD*/*ribN* expression in river water is similar to that in T medium (Additional file 2). Thus, river water seems to induce a general downregulation of riboflavin supply, while not inducing the overexpression of neither pathway.

**Fig. 3:**
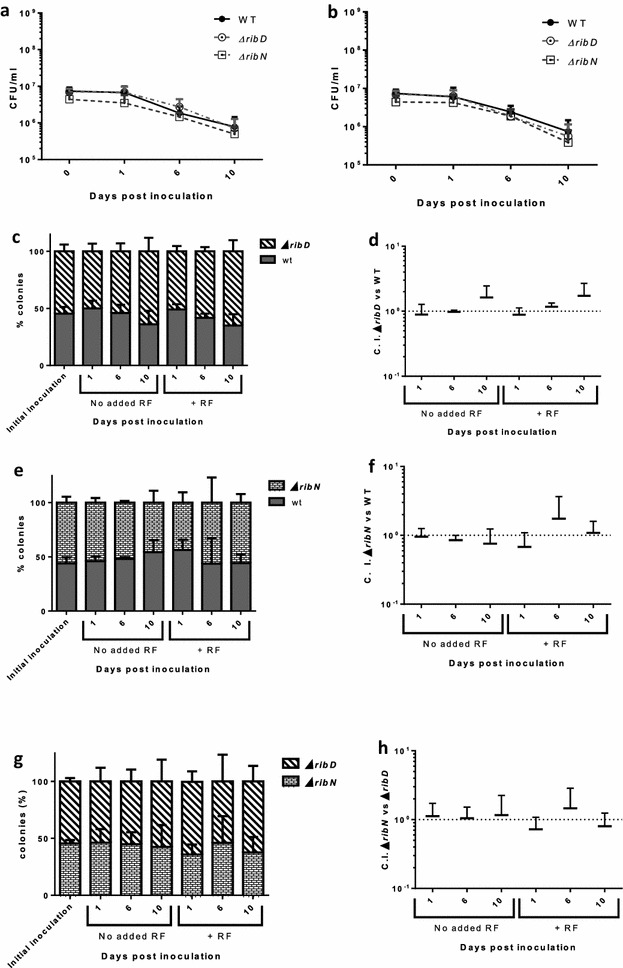
Culturability of *V. cholerae* in seawater. Filter-sterilized seawater was incubated with the indicated strains for culturability and competition assays determinations. **a** Culturability of strains in seawater without exogenous riboflavin. **b** Culturability of strains in seawater plus 2 µM riboflavin. **c** Culturability of strains in co-inoculation experiments of WT versus ∆*ribD*, WT versus ∆*ribN* and ∆*ribD* versus ∆*ribN* (**c**, **e**, **g**) and the same results expressed as C.I. (**d**, **f**, **h**)

When inoculated in seawater, the dramatic increase in culturability after 1 day of incubation observed in river water was not presented (Fig. 3a). Moreover, CFU were reduced at days 6 and 10. This pattern of culturability was similar for all three strains irrespective of the presence of exogenous riboflavin (Fig. 3a, b). In competition experiments, both mutants were recovered at the same levels as the WT (Fig. 3c–f) and also when competing against each other independently of the addition of riboflavin (Fig. 3g, h).

**Fig. 4:**
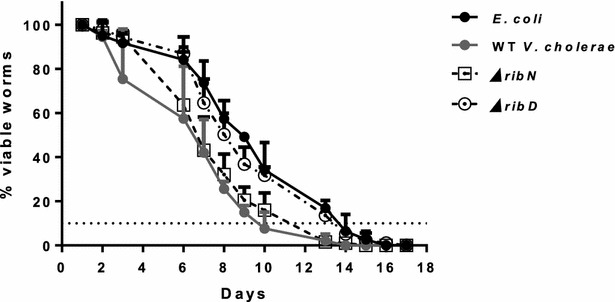
*Caenorhabditis elegans* lifespan in *E. coli* and *V. cholerae*. *C. elegans* were fed bacterial lawns of the indicated strains and survival determined by response to touch recorded. Results shown are the average and standard deviation of six measurements

Finally, we assessed the effect of the deletions on the lifespan of the *C. elegans* nematode. Feeding *C. elegans* with *V. cholerae* produces a moderate reduction in lifespan compared to the growth on *E. coli* OP50, the bacterial strain routinely used to feed *C. elegans* in the laboratory, i.e. the average time to reach 90% mortality was close to 14 days for *E. coli* but around 10 days for WT *V. cholerae* (Fig. 4). *C. elegans* growing on the ∆*ribD* strain had an increased lifespan, similar to when growing on *E. coli*, while the worms growing on ∆*ribN* had a similar lifespan to growth on WT *V. cholerae*.

## Discussion

The role of riboflavin transport in riboflavin prototrophic bacteria has started to be settled. In the riboflavin prototroph *Corynebacterium glutamicum,* the RibM riboflavin importer is able to provide riboflavin in the absence of endogenous biosynthesis, although the riboflavin intracellular levels are lower than in the wild type strain [13]. In this work, when growing in minimal media, exogenous riboflavin seems to fully replace the RBP but only at relatively high riboflavin media concentration (2 µM). This seems a rather large concentration for a transporter to achieve full provision of a metabolite in physiological conditions. This is probably due to low substrate affinity, co-transport requirements or other unidentified factor. This is hard to hypothesize because the transport mechanism for the RibN family has not been studied. Previous works have estimated the riboflavin concentration in open sea water in 30 pM and estuarine waters in a range of 45–128 pM [31, 32]. This is well below the 2 µM required to provide full growth to the Δ*ribD* strain in vitro. Thus, we hypothesize that 2 µM riboflavin is a concentration not likely found in environments where *V. cholerae* naturally thrives. Results obtained in river water indicate that endogenous riboflavin biosynthesis is both required and sufficient to maintain culturability of *V. cholerae*. In this condition, the strain lacking biosynthesis showed normal levels of culturability by day 1, but showed a decrease from day 3 onward. Prior to inoculation on river water, bacteria are grown in LB medium, which is plenty of riboflavin. In such conditions, it may be expected that bacterial cells of all strains contain high riboflavin intracellular levels. A possible explanation for the culturability pattern of the ∆*ribD* strain is that these vitamin levels may be sufficient for initial cell replication, but as time passes by and cellular riboflavin pools dilute, the strain unable to synthesize riboflavin starts to loose fitness. Notably, this result is analogous to that obtained by a previous study. While assessing the ability of a RBP-deficient *Brucella abortus* strain to survive inside macrophages, equal or even improved survival levels compared to the WT were observed at early time points. Later, survival dropped dramatically due to riboflavin depletion [33]. Strikingly, in river water 0.1 µM riboflavin was sufficient to restore culturability to the ∆*ribD* strain while this concentration only provided minimal rescue in T medium. This suggests than in some conditions, riboflavin transport is more efficient in fulfilling riboflavin needs. The analysis of the relative expression or *ribN* and *ribD* ruled out the possibility of an increased expression of the transporter in river water. Thus, it is conceivable that intracellular flavin needs may be reduced and/or that an alternative redox cofactor becomes available in river water. In these conditions, RibN confers an advantage to compete against the WT strain. This suggests that part of the riboflavin required by the bacteria may be provided by uptake. However, the fact that the strain lacking riboflavin biosynthesis was outcompeted by the strain deficient in uptake suggests that biosynthesis outweighs internalization in these conditions. In spite of this, the addition of riboflavin overturning this pattern reinforces the notion that when growing in riboflavin rich environments, transport may confer an advantage over biosynthesis. Notably, results obtained in similar experiments performed on seawater rendered a different outcome. Here, a clear reduction in culturability was observed for the three strains. Entrance of *V. cholerae* to VBNC state in filtered seawater has been reported before [25, 34]. Thus, in our experiment, *V. cholerae* likely also rapidly enters the VBNC state. As both mutants lost culturability at the same rate as the WT in single inoculations, it seems that elimination of *ribN* or *ribD* does not affect this process. The fact that similar numbers of each strain are recovered during competition assays does not necessarily means that the *ribD* and *ribN* mutants are successfully competing against the WT strain, but this outcome is probably related to the fact that all strains are rapidly loosing culturability in this condition. In order to see fitness differences, strains likely need to be in conditions allowing active growth. Nonetheless, further experimental evidence is needed in order to better depict the role of riboflavin provision pathways in seawater.

Finally, results in the host model suggest that riboflavin biosynthesis is specifically required for lifespan reduction on *C. elegans* while lost of *ribN* does not attenuates the bacterial potential of lifespan reduction. Given its general impairment to grow in riboflavin-restrictive niches, the increased lifespan on the ∆*ribD* strain may be due to a pleiotropic metabolic impairment rather than specific effects on virulence traits.

Overall, results indicate that riboflavin biosynthesis seems prevalent over riboflavin transport for fitness of *V. cholerae* in river water and on the *C. elegans* host model. Recently, a methicillin-resistant *Staphylococcus aureus* Δ*ribD* strain keeping an energy-coupling factor-RibU riboflavin uptake system was shown to require a low concentration of exogenous riboflavin to achieve wild type growth levels. This strain was not impaired in a murine model of host colonization, indicating that in such biological system, riboflavin uptake may fully substitute for biosynthesis [35]. Thus, it may be expected that the relative relevance of the riboflavin supply pathways varies across bacteria and their specific niches. In *V. cholerae*, riboflavin transport provides an improvement to compete in river water. Moreover, when growing in riboflavin-rich conditions, using exclusively the RibN importer offers an advantage over solely biosynthesizing riboflavin and this may be better accomplished in natural environments. This result indeed suggests that an incentive for uptaking over biosynthesizing exists.

## Additional files

**Additional file 1.** Expression of * ribD* and* ribN* is diminished in river water compared to expression in T minimal media. Expression of* ribD* and* ribN* in river water compared to expression in T media, assessed by RT-PCR as described in Methods. Media and standard deviation of three independent experiments are shown.

**Additional file 2.** River water does not changes the ribN/ribD expression ratio compared to growth in T minimal media. Relative expression of* ribN* compared to expression of* ribD *in T minimal media and river water as determined by RT-PCR. Media and standard deviation of three independent experiments are shown.
