# Supplementary figures and images for: Contribution of riboflavin supply pathways to Vibrio cholerae in different environments
Source: Gut Pathog. 2017 Nov 14;9:64. doi: 10.1186/s13099-017-0214-9 (PMC5686954; doi:10.1186/s13099-017-0214-9)

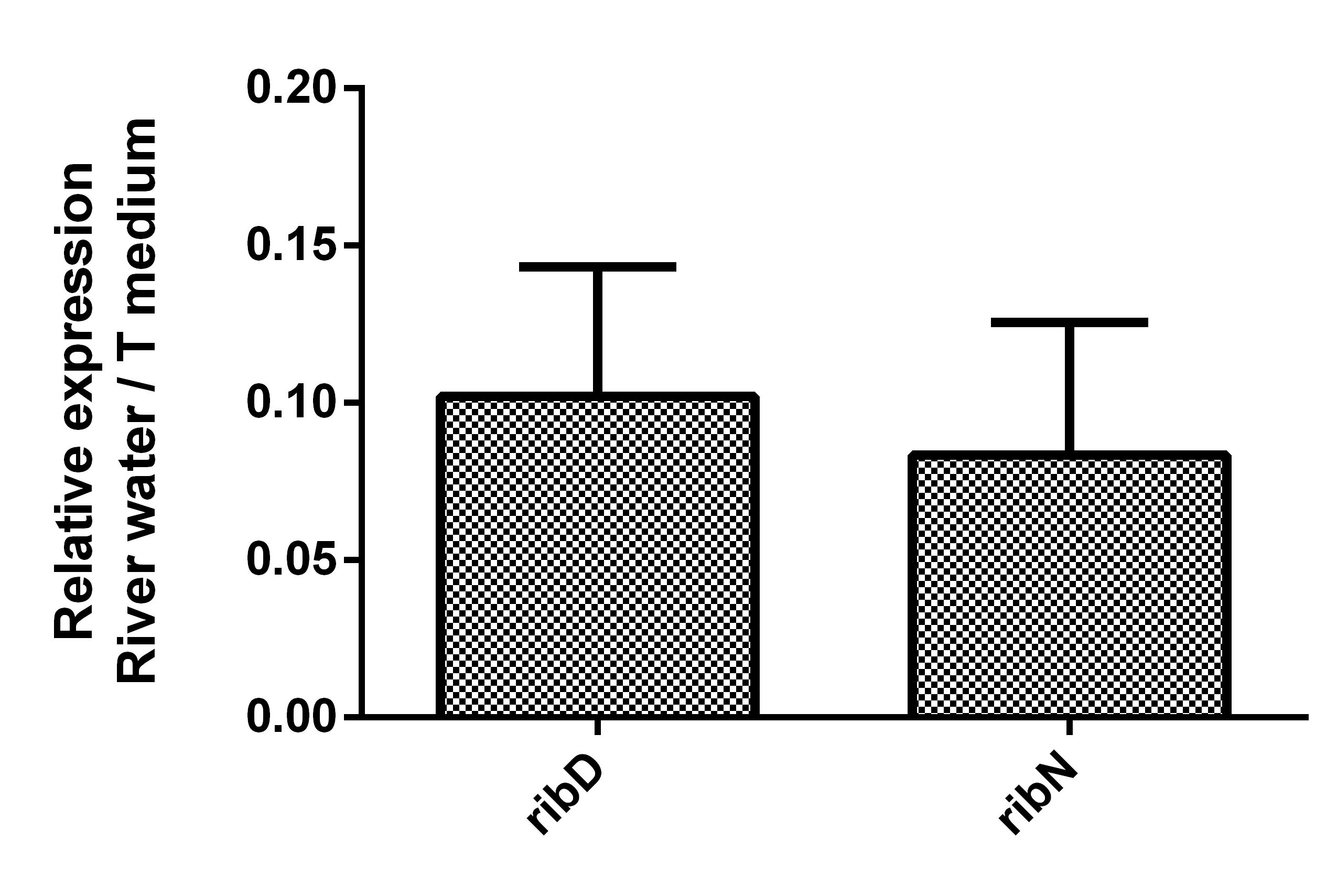

Supplement: Supplementary file 1 — Additional file 1. Expression of ribD and ribN is diminished in river water compared to expression in T minimal media. Expression of ribD and ribN in river water compared to expression in T media, assessed by RT-PCR as described in Methods. Media and standard deviation of three independent experiments are shown. [file 13099_2017_214_MOESM1_ESM.jpg]

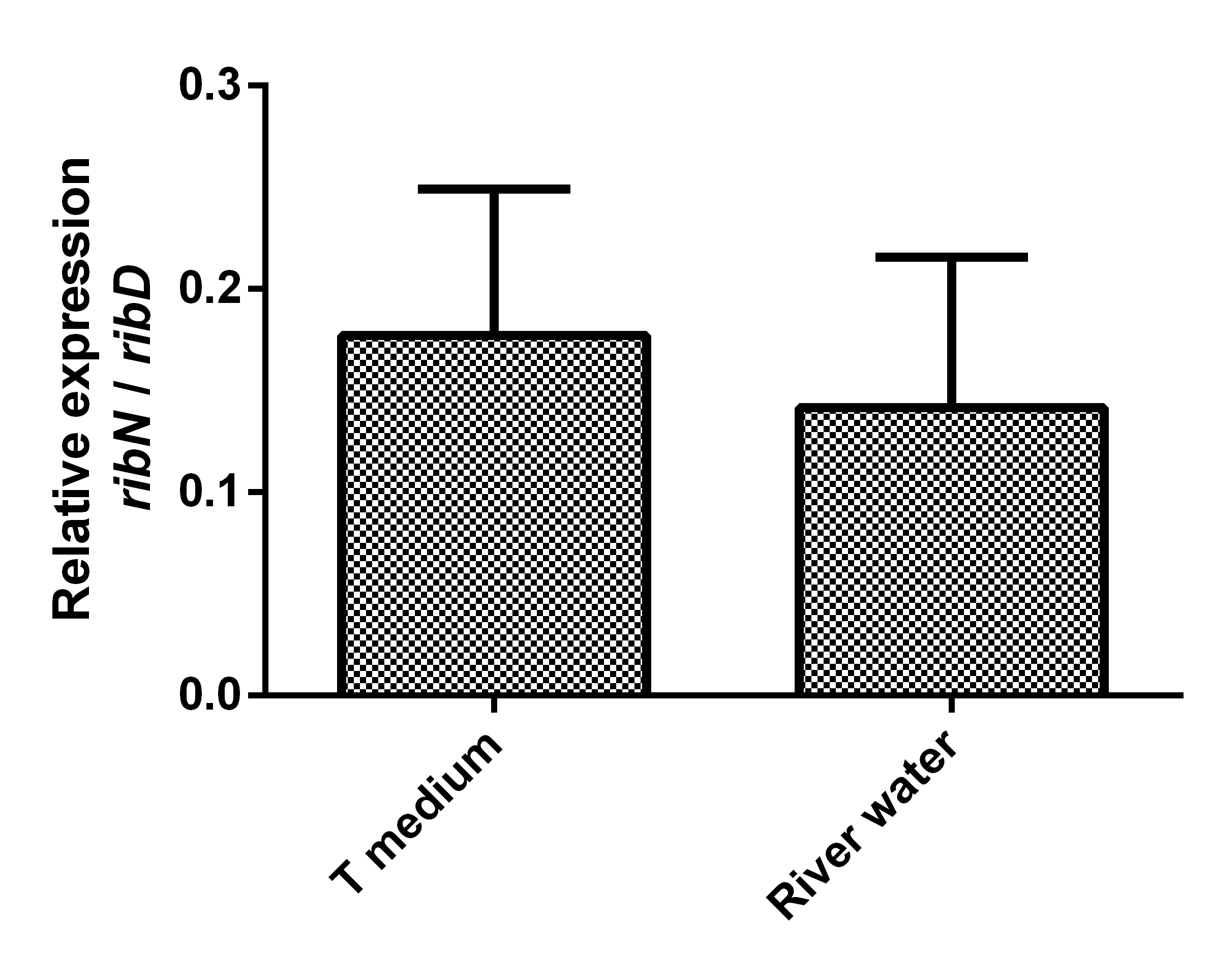

Supplement: Supplementary file 2 — Additional file 2. River water does not changes the ribN/ribD expression ratio compared to growth in T minimal media. Relative expression of ribN compared to expression of ribD in T minimal media and river water as determined by RT-PCR. Media and standard deviation of three independent experiments are shown. [file 13099_2017_214_MOESM2_ESM.jpg]
